# “Family Connections”, a DBT-Based Program for Relatives of People with Borderline Personality Disorder during the COVID-19 Pandemic: A Focus Group Study

**DOI:** 10.3390/ijerph19010079

**Published:** 2021-12-22

**Authors:** Isabel Fernández-Felipe, Amanda Díaz-García, José Heliodoro Marco, Azucena García-Palacios, Verónica Guillén Botella

**Affiliations:** 1 Department of Basic and Clinical Psychology and Psychobiology, Universitat Jaume I, 12071 Castellón, Spain; azucena@uji.es; 2 Department of Psychology and Sociology, Universidad de Zaragoza, 44003 Teruel, Spain; amandadiaz@unizar.es; 3 Departamento de Personalidad, Evaluación y Tratamiento Psicológico, Facultad de Psicología, Universitat de Valencia, 46010 Valencia, Spain; jose.h.marco@uv.es (J.H.M.); vguillenbotella@gmail.com (V.G.B.); 4 CIBER Fisiopatología Obesidad y Nutrición (CB06/03), Instituto de Salud Carlos III, 28029 Madrid, Spain

**Keywords:** borderline personality disorder, Family Connections, relatives, DBT, intervention, caregivers

## Abstract

The COVID-19 pandemic has had a significant impact on the family environment due to the difficulties that have been generated by job losses, deaths, increase rates of family and domestic violence, poor mental health outcomes, and estrangement in personal relationships. “Family Connections” (FC) is an internationally renowned DBT-based program that supports the families and caregivers of people with borderline personality disorder. The study took place at a Specialized Health Centre in Spain. A focus group with seven participants was organized for people who had previously attended an FC group. The participants were asked about their experiences during the confinement periods that was caused by COVID-19 as well as their experiences and opinions on relatives, skills practiced, their need to and the advantages of attending the group, and satisfaction with the FC group. The qualitative research web program Dedoose was used for the thematic analysis of the data. The results showed that the participants experienced various experiences during confinement; validation and radical acceptance were determined to be the most useful skills; the importance of professionals and the content as well as the sincerity of attendees and having a safe space were determined to be the greatest benefits of the programs; and the participants all indicated great satisfaction of the program. This study allowed us to explore the experiences of family members of people with BPD with their loved ones during the confinement period caused by the COVID-19 pandemic. We evaluated the use of the FC program skills in the family environment during confinement, and we analyzed the acceptability and satisfaction with the FC program.

## 1. Introduction

We are currently living in difficult times due to the global COVID-19 pandemic, a situation that has had a great impact on mental health due to the safety measures that have been implemented in order to prevent the spread of disease (physical and social isolation). These measures have resulted in loneliness and in a considerable reduction in social interactions, leading to risk factors for some mental disorders (e.g., schizophrenia and major depression). In addition, the uncertainty that surrounds the situation about the future as well as worries about health (one’s own or that of loved ones), give rise to or intensify fear, depression, and anxiety. The prolongation of these psychological problems can lead to serious mental illnesses such as panic, obsessive compulsive, stress, and trauma-related disorders [1,2]. One of the areas on which this virus has a great impact is families. The experiences of different families throughout the pandemic differ because many factors influence the relationship between well-being and COVID-19, including physical and mental health, politics [3], race and/or ethnicity [4,5], economics [6], individual and community resources [4], and country of residence [7]. Studies show that pandemics have a direct effect on people’s well-being, creating problems such as illness, economic instability, and emotional isolation, among others [8]. To mitigate the spread of the pandemic, many countries, including Spain, were completely confined for more than two months. This great effort on the part of the population had a strong impact on stress, depression, fear, anger, boredom, and stigma [9]. In addition, a study by Vindegaard and Benros [10] shows that psychological well-being in adults has declined compared to periods during the periods before the emergence of COVID-19.

The family environment is one of the areas that has been the most affected. The COVID-19 crisis has had a great impact on families because many of them have been fighting against health threats and difficult family situations such as family and domestic violence. Unfortunately, many people have directly experienced the loss of family members, which has led to deep sadness, anxiety, and homesickness, among others [11]. In addition, there are also indirect effects of the pandemic that have arisen due to the set of limitations that have implemented in terms of interaction with the outside world and due to the intense moments that have been experienced in the family environment. Some of the most vital restrictions were those that were placed on physical and emotional contact, which in many countries limited to people living together. This restriction has had a significant impact, giving rise to strongly shared processes that provide many possibilities for both benefits and disadvantages [12]. In addition, a study by McFarlane [13] suggests that family difficulties arise for caregivers who have dealt with a family member with the help of others who are no longer present, as in the case of caregivers of people with mental disorders [14].

One of the most complex mental disorders is borderline personality disorder (BPD). It is characterized by high emotional intensity and instability as well as impulsivity and is associated with high rates of self-harm and suicide, with these two behaviors being seen in 69–80% of the BPD population [15]. In addition, this problem is associated with high rates of 24 h hospital intervention, recurrent use of health services and, consequently, high associated financial expenditure due to the use of these services [16,17,18,19], including the use of emergency services and multiple professionals [19,20,21]. It is a major public mental health problem that causes great distress to both patients and their loved ones [22].

BPD causes challenges among individuals and their families [22]. Thus, it is important for both patients and families to receive specialized care and psychological treatments based on empirical evidence. Maladaptive family communication patterns play an important role in the etiology and maintenance of BPD. The family members of individuals with BPD are more likely to develop psychological problems [23,24], and one consequence is the perceived burden on caregivers [25,26,27]. On the one hand, some studies show that misinformation and uncertainty about their family member’s diagnosis and the progression of the disorder increase levels of burden and depression [28,29]. On the other hand, studies suggest that when family members are part of the treatment process for people with BPD, relapses are reduced, recovery is easier, and the quality of family life improves [29,30].

Fortunately, group intervention and skills training programs for the family members of people with BPD exist and are empirically supported. Almost all family skills training programs are based on Dialectical Behavior Therapy (DBT) or DBT adaptations [31]. DBT [32] is a specific psychological treatment for people with borderline personality disorder that addresses the symptoms of behavioral and emotional dysregulation that often materialize as suicide and parasuicide. DBT belongs to the group of so-called third generation therapies and incorporates the cognitive and behavioral approach, emphasizing the context and function [33]. The program for the family members of people with BPD that has the most empirical support is Family Connections (FC) [34]. FC consists of six modules that are divided into two sessions each, and each module has specific aims and practical exercises. The modules are the following: introduction, family education, relationship mindfulness skills, family environment skills, validation skills, and problem management skills.

Five uncontrolled clinical trials with pre- and post-treatment and follow-up assessments [24,34,35,36,37] have been conducted to date. The results of this program have been replicated, and the results were consistent and maintained or improved over a 3- or 6-month follow-up period. Studies show significant decreases in burden, grief, anxiety, and depression, and significant increases in the participants’ subjective experiences of mastery, empowerment, well-being variables, and family functioning [31]. These encouraging results for family climate and functioning may be explained by program content that validates the patients’ coping behaviors, decreases their psychological symptoms, improves well-being and relationships between family members and patients, leads to a greater understanding of the problem, works to reduce the stigma of the disorder, and increases family empowerment [37].

Qualitative studies are an interesting method that can be used for collecting and analyzing non-numerical data to understand concepts, opinions, or experiences. A qualitative study with eight family members of people with BPD by Dunne and Rogers [38] showed that these family members are poorly served or not served at all by mental health services and that they need support from professionals to improve their well-being. Another qualitative study of 19 family members of people with BPD used open questionnaires and group interviews. The results determined that the relatives are continually afraid that something bad might happen, and they try to keep the family atmosphere as bearable as possible in addition to feeling guilt and lifelong grief about their relative with BPD. In terms of mental health services, the family members of people with BPD feel left out and abandoned, and they have lost confidence in mental health professionals [39]. Kay, Poggenpoel, Myburgh, and Downing [40] conducted a qualitative, exploratory, descriptive, and contextual study with eight family members of people with BPD, and the results showed that these relatives have a lack of knowledge about the disorder, which produces feelings of disempowerment. Finally, another qualitative study on the experiences of family members of people with BPD with self-injurious behavior and attempted suicide was carried out with four family members [41]. The results showed that these relatives suffer from chronic and traumatic stress as well as a strain on the family climate and between the relatives and mental health services. These results indicate the need for an approach that considers family members in a meaningful way in treatment as well as in their relationships with mental health services [41].

These results suggest that qualitative research allows us to acquire more detailed and richer information in the form of descriptions and to observe the context and social meaning and how they affect individuals. In addition, communication takes place in a more horizontal way through the use of different tools that allow for unexpected discoveries to be made during the research process. Qualitative research also makes it possible to study individual experiences in greater depth. Given the findings from other qualitative studies with family members of people with BPD, it is important to explore the views and experiences of these people in the extraordinary situation of the COVID-19 pandemic.

This research has several objectives. The first objective was to assess the experiences that the family members of individuals with BPD have had with their loved ones during the confinement period caused by the COVID-19 pandemic. The second objective was to evaluate the use of the FC program skills (validation, radical acceptance, emotion regulation, problem management, and mindfulness of relationships) in the family environment during confinement. The third objective was to evaluate the acceptability and satisfaction with the FC program.

## 2. Methods

### 2.1. Participants and Recruitment

The participants were recruited from a group that received FC prior to and during COVID-19 confinement. These groups were previously formed by randomizing a sample of relatives of people with BPD who had been recruited for another efficacy study. They received the FC program for three months, and the follow-up coincided after the confinement, which is when we conducted this qualitative study. The selected family members received a phone call from our research group, inviting them to participate in the focus group if they had no contact with a person who was positive for COVID-19, had no symptoms, and were not waiting for the results of a diagnostic test for COVID-19. This group consisted of nine family members: four mothers, two fathers, a partner, and two children. In all, there were four families. All of the participants attended over 80% of the program sessions, but only seven of them attended the focus group after confinement.

Four families composed of seven family members of people with BPD were selected. They stated that they complied with the established rules, and they presented their motivation for participating in the focus group discussions. Prior to the focus group, the family members were asked to sign a consent form allowing the researchers to write down the content of the discourse in the focus group discussions and to publish the information that was collected. They were informed that the presentation of the data would be confidential and that no statement would be able to be traced to a particular participant.

### 2.2. Description of “Family Connections”

Family Connections is an intervention program that is based on DBT strategies. It is composed of six modules that are divided into two sessions each, and it was created to improve family attitudes and to reduce family exhaustion [34]. The modules are (a) Introduction, which provides information about the aims of the program, the criteria and symptoms of BPD, and the role of emotion regulation; (b) Family Education, which provides information about treatment programs for BPD, comorbid disorders, the biosocial model, and the transactional model of the development of BPD; (c) Relationship Mindfulness Skills, which presents states of mind, emotion regulation skills, and mindfulness of the relationship; (d) Family Environment Skills, which explains radical acceptance, and the aim is to understand the relationship between the individual and the family’s welfare and the importance of maladaptive ways of thinking that are related to blame; (e) Validation Skills, which presents validation and self-validation skills as well as learning how to set clear limits and how to achieve self-respect; and (f) Problem Management Skills, which focuses on interpersonal efficacy, defining problems and solutions, and problem management skills. Each module has specific objectives and practical exercises as well as videos with examples of people with BPD and their relatives.

### 2.3. Data Collect and Procedure

A qualitative method was used in this study. A focus group with seven participants was organized in July 2020 for people who had previously attended a FC group. The participants simultaneously participated in a single two-hour session with the researcher, and they completed a questionnaire with open and closed questions. The discussions in this focus group took place in a large and safe place that allowed all the COVID-19 security measures to be respected and was located in a Spanish center that is specialized in personality disorders. The organization of this focus group was motivated by the fact that this would allow contact after the COVID-19 pandemic confinement among the relatives who attended the FC group. Before and during the pandemic, a face-to-face skills group was conducted with the relatives of people with BPD, which also used the FC program, where they learned DBT-based strategies. One researcher (IF-F) asked all of the study questions during the session and moderated the discussion. The participants responded to the questions that were asked, interacted with each other, and listened to other the responses of other participants. The focus groups were held face-to-face in the clinic and lasted two hours. The focus groups were carried out by two researchers (IF-F and AD-G) and were transcribed verbatim by the first author.

### 2.4. Ethical Considerations

The guidelines of the Declaration Helsinki and existing guidelines in Spain and the European Union for the protection of patients in clinical trials were followed in this study. The Ethics Committee of the University of Valencia (Valencia, Spain) approved this study. The trial was registered at ClinicalTrial.gov under trial number NCT04160871.

### 2.5. Measures

#### Interview Protocol

A semi-structured interview with open-ended questions was designed that focused on (a) the experiences that caregivers had during confinement with their relatives; (b) the skills that they learned during the program, which skills were the most useful to them, and which skills they used during the confinement; (c) their needs before attending the therapy group, the advantages of attending it, and adherence to the group; and (d) satisfaction with and acceptance of the FC group. For this interview, the construction process included an initial discussion among the team members. Second, the questions were elaborated by two research team members separately. Finally, agreement was reached by comparing the two lists of questions, trying to balance the greatest number of topics with the least number of questions.

Examples of questions that were addressed in the focus groups are provided in Table 1.

Opinion of treatment scale by modules (OTSM). The Opinion of Treatment Scale by Modules is an instrument that was developed by our research team and that was adapted from Borkovec and Nau [42]. This questionnaire evaluated opinions that participants have about and acceptance of the program using numerical scales and open questions as well as their acceptance towards any changes made to the six therapeutic modules. The questions are related to the logic of the treatment, the degree of satisfaction with the program, whether they would recommend the program, and the usefulness and expectations of the program. In addition, they assessed the learning and usefulness of the skills that had been taught in the module on a scale from 0 (none) to 10 (high).

### 2.6. Data Analysis

The qualitative analysis of the focus group was conducted using a qualitative research web program called Dedoose, which is a qualitative research program that contains tools to manage and analyze data that have been obtained from qualitative information. First, a separate set of codes was created in coding themes by an expert from the research team with expertise in qualitative studies. We used the Consolidated Criteria for Reporting Qualitative Research (COREQ) [43] for the coding of the focus group transcript and for the analysis of these data. To perform the content analysis, we relied on the research literature in this field, and this resulted in the themes that appeared in the coding process [44]. Induction and deduction methods were used for the data coding process. A double-blind design was used to conduct the coding by two researchers independently. This analysis addressed one of the main points of the focus group: the experiences of family members of people with borderline personality disorder during the confinement period that was induced by COVID-19 and the use of the skills that were learned in the Family Connections skills training program.

## 3. Results

### 3.1. Characteristics of the Relatives and Patients

Seven family members participated in the focus group. The participants were three mothers, two fathers, a daughter, and a husband of a person with borderline personality disorder. None of the participants were in any type of ongoing therapeutic program at the time of the qualitative study. The sociodemographic characteristics of the relatives and the patients are shown in Table 2. All of the fragments of the interactions between the relatives were in Spanish and have been translated into English.

#### 3.1.1. Family 1

The family members in Family 1 consisted of the patient’s partner and daughter. These two relatives had been given multiple diagnoses during their relative’s journey through the health system, and they had never learned management skills that could be used with their relative or had received psychoeducation about BPD. The patient was a 57-year-old woman who was on leave from work. She had a diagnosis of bipolar disorder and BPD. She had been suffering from the disorder for more than 30 years, with numerous hospital admissions and two suicide attempts. She was in a state of high impulsivity.

#### 3.1.2. Family 2

Family 2 consisted of the patient’s father and mother. Because the patient’s diagnosis was recent when they started the program, these relatives had never received treatment or had been informed about the persons diagnosis. The patient was a 28-year-old woman who studied and worked at the same time. She had a diagnosis of BPD. She had been admitted to hospital because of a suicide attempt. The characteristics of this patient were high emotional and behavioral dysregulation. She had learned to regulate these areas, and she was in an advanced stage of treatment, meaning that she had completed the DBT program twice and was no longer receiving treatment.

#### 3.1.3. Family 3

Family 3 consisted of one mother. This mother had attended several therapeutic groups for family members and had received psychological treatment. She had accompanied her daughter during the psychiatric and therapeutic process for 20 years. The patient was a 37-year-old female university student. She had a diagnosis of BPD and Anorexia Nervosa. She had had multiple hospital admissions due to three suicide attempts, one of which had a high risk of lethality resulting in irreversible physical injury. She had sequelae from that autolytic attempt and was in intensive psychological treatment.

#### 3.1.4. Family 4

Family 4 consisted of a mother and a father. Due to the patient’s recent experience in the healthcare system, these relatives came to the group confused about the diagnosis. They had never participated in a group of relatives or received psychological treatment. The patient was a 22-year-old female university student. She had a diagnosis of BPD and Major Depressive Disorder. She had never had any hospital admissions or suicide attempts. The main characteristics of this patient were a low mood, emotional dysregulation, and identity dysregulation.

### 3.2. Qualitative Results

The results that are shown are from the experiences of four families (Family 1, Family 2, Family 3, and Family 4) during the confinement period that was implemented due to COVID-19. The responses could be divided into five themes.

#### 3.2.1. Theme 1: The Impact of COVID-19 Confinement on People with BPD

Spain is one of the countries that has been the most affected by the COVID-19 pandemic [45]. One of the restrictions that had the greatest impact is the total confinement of the population for more than two months. All of the relatives in this study live with people with BPD. Therefore, all of the experiences in this section refer to having spent the confinement period together. The experiences of these four families were very different due to where each patient was in their treatment process, among other factors.

##### Positive Experiences

Family 1 reported good experiences during the confinement period, and the daughter said:

*“In general, we were afraid to be at home to see what could happen because we of course didn’t know how we would react, and they are much more sensitive […], so there has been a little bit of tension and nervousness in that area, like a punching bag… and I tell her, but Dad is taking it personally, she has a disorder, and when she’s up here she lashes out at the person closest to her, which is you, because you’re stuck with her 24 h a day. But she was fine with us, with everything that was happening. I believe she has acted very well, was strong, and helped me in everything; she has been incredibly positive.”*

Family 2 also had a good experience, and the father commented:

*“We’ve been amazed. Wonderful, very surprising, we could not believe it. I had a hard time because my wife got the coronavirus. Then, the whole family was followed up with, and they did the diagnostic test, the serology on all of us, and the only one who had really gone through it is her, not us. One day she was overwhelmed, and she said to her brother “please, I want to rest, I am studying”. She was studying non-stop every day; she has signed up for a lot of online courses, and she has done everything. She is very happy. And there is more. Two weeks ago, suddenly she became independent”.*

##### Neutral Experiences

In contrast, the mother from Family 3 commented that it was *“Strange, a very strange thing, I don’t know,”* because neither she nor her relative assimilated to what was happening, and they were very irritable about any little thing.

##### Negative Experiences

Finally, Family 4 did not have a good experience with their daughter during the confinement, although, as the mother said, it was not all negative:

*“I don’t even feel like saying anything. Because everybody is good… and we are not. We had improved a lot just a few weeks ago, and we have gone backwards. During the confinement, it has been… uf. Sometimes, it’s hard for us to even know about allowing yourself to lose control because I don’t know if I allow myself to or if I lose it without permission because I’m tired; I’m very tired. You are more dependent than you were before the confinement. We have achieved something good because we also have to say something good, and that is that the social isolation she had is gone because now she stays with her friends”.*

#### 3.2.2. Theme 2: Learning and Knowing What Is Going on with Their Relatives

The family members learned DBT skills in FC in order to know about and accept the problem their loved one has, empower themselves, improve the family climate, and enhance their quality of life. The partner in Family 1 opened the topic by saying *“We have learned things that we may not have known how to manage”* because none of the participants had previously participated in a skills group for family members of people with borderline personality disorder. The daughter in this family summarized what she learned in terms of both psychoeducation and skills:

*“I think that the most important thing we have learned is to know what our family member suffers. I think none of us knew because we could not understand why they behaved as they did. Learning about their problem and putting into practice all the methods to improve our coexistence with them has been positive because there came a time when we could not live with that person.”*

Regarding Family 2, the father commented that he had learned to set limits, something he did not do before attending the group for fear of triggering a crisis:

*“Look, one of the things we have learned is that I overprotected my daughter out of fear that a crisis would occur. But there came a moment, when I came here to the group, that I said, “this is as far as I go”. I was doing it for her sake, but then I realized that I was being very selfish, and I felt guilty, and I have learned all that here, not that I didn’t know it, but to say, “it’s not just me who is thinking it, it’s that they are telling me”. In short, I have now learned to say “No.”*

The mother in this family referenced one of the objectives of the program:

*“It is like the famous statement: “let’s take care of the caregiver”. If we don’t take care of the caregiver, they won’t be able to take care of you because you are sick, you can’t take care of them because you don’t know how.”*

Finally, the mother in Family 4 referred to all of the participants in the group and their motivation for coming to learn how to manage the relationship with their loved one:

*“It seems to me that here we all worry a lot, you with your mother, you with your wife, you with your daughter, and you are of course and another relative who is not here today. I think that we have come here because we are eager to learn and to know what is going on with our relative. We have taken in everything to learn.”*

#### 3.2.3. Theme 3: Validation and Radical Acceptance Were the Most Used and Useful DBT Skills during Confinement Due to COVID-19

The participants were asked about the DBT-based skills that they learned during the FC program and that they had to perform during the confinement period. These questions were categorized into (a) most useful skills and (b) the skills that were the most used during the confinement period.

##### Most Useful Skills

Of all the skills that the FC program teaches, all of the family members responded that the most useful ones were validation and radical acceptance:

*On the one hand, the partner in Family 1 said, “I think acceptance has been the most important thing because it helps you realize what you have and what you must accept, and that’s how it is. Not hitting the wall.”*

*On the other hand, the mother in Family 4 commented that “Validating her feelings, her sensations, and all that has seemed very important to me. Knowing how to say, “I understand that you are like this…”*

In addition, the psychoeducation prior to the skills seen in the program helped them to understand their relatives’ diagnosis.

##### Most Used Skills during the Confinement

As for the skill that was the most used during confinement, all of the family members responded that it was validation. This is one of the skills that surprised them the most during the program, and along with the multimedia material, they integrated this concept very well. In addition, they saw significant changes when they started using it. The daughter of one of the patients stated that due to the large amount of time they spent at home because of the COVID-19 restrictions, she had many aspects to validate. The skills that were used the most often by the family members can be seen in Table 3.

#### 3.2.4. Theme 4: Professionals, the Content of the Program, the Sincerity of All the Attendees, and Having a Safe Space

One of the problems that arises during the psychological treatment of people with mental disorders is that the caregiver of the family member with BPD is often neglected. The participants in this group commented that they had been accompanying their family member to therapy for many years, but they had never had the opportunity to be part of a family-to-family support group and receive clear and comprehensive information about the problem their family member was having and skills for dealing with it. When asked why they came to the group, the answers were the following:

*“To learn, learn how to handle the situation”(mother in Family 3); “To know how to act because we did not know how to act on many occasions”(father in Family 2); “We are in a situation that is a borderline situation, and I have to find a way to cope with it” (partner in Family 1); “I think that it’s a good thing that we all came here with an empty glass, with a blank slate, and that we all came here to fill it up” (father in Family 4); “We came here to learn”(mother in Family 3); and “Because we were lost” (mother in Family 2).*

Many times, in therapeutic groups, there are participants who do not start the group or who drop out. All of the participants who attended the focus group attended more than 80% of the sessions. However, as participants in a therapeutic group, they were asked why it is necessary for members to join the group.

The most frequent responses were the education that was received from the professionals who created the group and the help they provided, the content of the program, the sincerity of all of the attendees, and having a safe space to express their concerns and worries. The mother in Family 4 said:

*“Knowing that we are not the only ones and that, as you said before, we are not doing so badly… Because all of us here, I imagine, have been told so many diagnoses for our relative and so many strange things that now it turns out that there is a diagnosis that fits well “.*

That is, the group is necessary to provide clear and concrete information about the problem and to form a support network among equals where they feel listened to and supported. The father in Family 4 said

*“…that we have seen that we share many things in common with others, and one very important thing I think is that they listen to you and help you and support you and each other and you listen to others”.*

Relatives of people with BPD suffer, among other things, from the burden of their family member. The father in Family 4 added:

*“It is common that we have someone very close to us who causes conflict. Knowing that he or she is not alone in the world relieves you of a lot of weight. It frees you from the burden, and then the capacity that each one has to transform it or to be able to contribute to that family member, that is already inside of you, but to be able to communicate and to be able to say it in public”. *The mother in this family interacted with him, adding *“It’s just, who do you tell your problems to? No one, you can’t,” and the father in Family 2 replied, “Because people don’t seem to understand you”.*

In addition, the father in Family 4 mentioned one of the benefits of the group’s privacy and sincerity:

*“And another thing that I think is good, I don’t know if any of you have thought about it, but what I have thought about is the fact that here everyone belongs to different places and backgrounds and outside of here we don’t have any relationship, none, and that’s positive. Why? Because when you and I come here, I come to tell you about my daughter’s problems and my problems with her, but they stay here; they do not leave here. Therefore, I can see them around the city one day, and I will say hello to them. Moreover, for me that is fundamental, the fact that you come to a group of people that you do not know, and you are willing to come. I was in another city today, and you have to come from work and leave your work and come here. You share intimate things, and they stay here, in the sense that if I, for example, knew you from before, it would be more difficult. I would not be so open.”*

Finally, another advantage that the group provides is the increase in hope about their loved one. The mother in Family 4 said:

*“We’re going to leave with hope because when we came here, we didn’t have any, at least not us”.*

#### 3.2.5. Theme 5: Great Satisfaction with and Acceptance of the FC Group

As for the satisfaction with the program and the support from the group, all of the participants responded that it was great. In addition, they said that the lack of knowledge about their relative’s diagnosis and the lack of tools and skills made them feel lost. The daughter in Family 1 commented:

*“It has been very good for us because we were lost, and it has helped us to realize that it is something that affects many people, and that the reactions of our relatives are similar.”*

In addition, the fact that it was a safe environment where they could interact with each other gave them a lot of satisfaction. The mother in Family 4 said:

*“Then we come here, and we have something in common, we share. I also find it very enriching that we can talk to each other. A member of the group could have seen something that works that I may not have seen.”*

The partner in Family 1 replied:

*“The first time I came here, I was a little reluctant because had to expose my problems and speak in public, but as the sessions went on, I thought “I’m looking forward to it, because I want to express this, and I want them to know it”.*

In addition, the mother in Family 4 commented:

*“You feel sheltered.”*

Finally, the father in Family 4 said:

*“This is like you go and say things that in other places we can’t. You open, you tell, and it is a good experience. It is therapy for the caregiver. It is learning how to take care of ourselves so that we can take care of them later.”*

The satisfaction with and acceptance of the FC program bythe family members can be seen in Table 4.

## 4. Discussion

The aim of this study was to explore the experiences of family members of people with BPD with their loved ones during confinement period that was implemented due to the COVID-19 pandemic. Furthermore, we evaluated the use of the FC program skills in the family environment during confinement, and we analyzed the acceptability and satisfaction with the FC program. Five relevant themes emerged: (a) various experiences of family members of people with BPD during confinement due to COVID-19; (b) learning and knowing about the experiences of their relatives; (c) validation and radical acceptance were the most used and the most useful DBT skills during confinement due to COVID-19; (d) professionals, the content of the program, the sincerity of all the attendees, and having a safe space were considered to be significant benefits of the program; and (e) the participants demonstrated a great level of satisfaction and acceptance of the FC program. Although these issues are linked to ideas from previous studies [38,39,40,41], this is the first study to describe the experiences of family members of people with BPD and to explore the use of the FC program during confinement due to COVID-19 as well as analyzing the satisfaction with and the acceptance of the FC program.

The family members of people with BPD needed to talk about their experiences during confinement due to COVID-19. There were positive, neutral, and negative experiences with their relatives. Some of them mentioned how surprised they were that everything was going well in their family and how well they were coping with these difficult and uncertain times. However, others commented on how difficult it was for them to live with this family member and the setback in the symptoms that was seen during the confinement. Studies suggest that when family members are part of the treatment process for people with BPD, relapses are reduced, recovery is easier, and the quality of family life improves [29,30]. Sharing these experiences and interacting with other family members with similar problems and with professionals provides a network of support and a feeling that they are not alone.

One of the most important things that the family members learned during the program was what the family member diagnosis means and helped them to understand their behavior was well as how to practice the tools that could be used to increase family functioning. In addition, an important aspect that they verbalized repeatedly is that they were now aware that the caregiver must take care of him or herself in order to provide good care for their family member. This agrees with the line of results found in other studies that have suggested that misinformation and uncertainty about their family member’s diagnosis and the progression of the disorder increases relatives’ levels of burden and depression [28,29].

As for the skills that were provided by the FC program, during the confinement, validation was the skill that was used the most often. This is one of the skills that surprised them the most during the program, and along with the multimedia material, they integrated this concept very well. In addition, they saw significant changes when they started using it. Another skill that they found quite useful was acceptance because it helped them to release the burden and grief related to having a family member with BPD and allowed them to stop becoming frustrated about something that was not under their control. In line with the literature, creating or maintaining a safe and validating family environment in which all family members are accepted can be very difficult when one or more family members are persistently distressed by the possibility of their loved one committing suicide [32]. One of the reasons for the creation of FC was to improve validation and acceptance skills to create and maintain a validating family environment in the face of crises [34].

One problem that arises during the psychological treatment of people with mental disorders is that the family member is often neglected. The participants in this group gave a lot of importance to the training of the professional who oversaw the group, the content of the program, the sincerity of all those attending the group, and having a safe space where they could transmit their concerns and doubts. In one study, Hoffman et al. [34] described the “surplus stigma” that the family members of people with BPD experience due to a lack of understanding and prejudice towards people diagnosed with this personality disorder. They point out that these attitudes are not only related to society, but they also stem from the healthcare system. This was also reflected in our study, where the participants mentioned the importance of feeling safe in a non-judgmental space where they could talk about complicated issues without fear of prejudice and stigma about this disorder.

The families who participated in this focus group mentioned that they were very satisfied with the FC program and that the information about their family member’s diagnosis and the skills that they learned made them feel safe. In addition, belonging to a group whose members have something in common and who have lived through similar family experiences made them feel that they were not alone, that they could be hopeful, and that they were listened to. We can conclude that the FC program is satisfactory for family members of people with BPD and that it generates security due to the availability of updated information about the diagnosis of their loved one and the learning of different skills that can be used to manage the family situation. Furthermore, we conclude that it generates an emotional support network where family members feel listened to by others and makes them more hopeful.

## 5. Practical Implications

This study suggests that FC is a good skills program for family members of people with BPD. In addition, it shows that it is crucial for family members to acquire knowledge about their relative’s diagnosis and to create a support network of people with similar problems where they can interact and listen to others. Unfortunately, the family members of individuals with BPD are very affected, and they often experience high levels of anxiety, stress, burden, and hopelessness [34]. Therefore, family members need to be supported by both professionals and other family members, and this support and recognition should be promoted in the mental health network and in campaigns. Some international scientific associations have issued statements on how to accommodate the consequences of the pandemic. Stewart and Appelbaum [46] state that a COVID-19 diagnostic test should be performed on those patients presenting with symptoms and, if positive, they should be isolated in specialized patient units. However, such isolation cannot violate human rights or neglect the patient’s treatment needs. In addition, they must be attended to virtually and, if this is not possible, all public health protocols must be carried out in person.

Another practical implication is that FC can help families with a BPD relative to cope, not only with life in general, but also with extraordinary events, such as being confined at home due to a pandemic. It is worth exploring whether the skills that were learned during the FC could also help other families with relatives with other psychological or physical problems to cope better with stressful events.

## 6. Strengths and Limitations

This qualitative study allowed us to acquire detailed information about these caregivers’ experiences with their family members with BPD during confinement due to COVID-19 through the descriptions that they provided of their experiences from a more social context that allows us to understand how these experiences have affected them. In addition, thanks to more horizontal communication, interactions and responses emerged during the focus group that were not premeditated and thus provided very rich information during the research. Another strength of this study was the contribution to research in this population using a qualitative method, which is still very scarce in relatives of people with BPD.

This study has some limitations. The main limitation of the study was the small sample that we were able to obtain after the COVID-19 period. Larger samples are more representative of results. Another limitation was that only one focus group was conducted and that people with BPD did not participate, giving us other valuable information. In addition, the fact that the study was only conducted in a health care setting in Spain limits the generalizability to other parts of Spain and internationally. Another possible limitation is that this study only really captures the experiences of families who are still in good relationships with the person with BPD and not those of people who are the relatives of an individual with BPD wo is no longer receiving support. The contents of the results depend on the willingness of the family members to disclose this information as well as the ability of the interviewer, one of the key points in qualitative studies [47]. Another limitation could be that one of the participants in the FC program did not participate in the focus group and could have provided relevant information for this research. Despite these limitations, this is the first study to describe the experiences of family members of people with BPD and to explore the use of the skills that were learned from the FC program during the confinement period that was implemented due to COVID-19 in addition to analyzing the satisfaction with and acceptance of the FC program, which could be useful in designing and implementing interventions for family members. The focus group was a safe space for these family members to discuss their experiences and to express themselves, allowing them to ask questions to the professional and to interact with other family members.

However, we hope that future generations of family members will have greater access to the FC program and that they will be able to enjoy and take advantage of the possibilities that are offered by this skills program. It is fairly limited in terms of the costs that are involved in accessing the program due to the lack of economical and human resources in the Spanish public sanitary system. It is recommended that this program become more accessible through the reduction of those barriers.

## 7. Conclusions

We can conclude that Family Connections is a skills training program for family members of people with BPD that has both clinical and family environment benefits. Although there have been several studies on the effectiveness of the program, it is necessary to listen to family members and to consider how they live their daily lives with their loved ones. In addition, we know that skills practice is often complex, even more so when living in a context of confinement due to a global pandemic. It is necessary for health professionals to be trained in skills training programs, as this cost can be offset by the improvement in the well-being of families as well as in the reduction of psychological symptoms and the burden on the family environment. Finally, further research with this population and the implementation of these highly accessible family groups is needed to reach as much of the population as possible.

## Figures and Tables

**Table 1:** Questions ^1^ addressed in the focus group.

How was the confinement and what experiences did you have with your family member in this period?
What of the skills that have been used in the program have you learned, and which have been most useful to you?
What skills have you used the most during confinement?
Why did you come to the FC group?
What experiences have you had with the group?
What do you think is essential for a group like this to work?
What advantages have you found from attending this group compared to your usual treatment?
Why do you think it is necessary for family members to adhere to this group?

^1^ Translated from Spanish into English.

**Table 2:** Characteristics of the relatives and patients.

Participant	Characteristics	Mean (SD)
Caregiver	Age (years)	53,43 (27 to 68)
		n (%)
	Sex	
	Female	5 (71,4)
	Male	2 (28,6)
	Relationship with the patient	
	Mother	3 (42,9)
	Father	2 (28,6)
	Husband	1 (14,3)
	Daughter	1 (14,3)
Patient	Age (years)	36 (22 to 57)
		n (%)
	Sex	
	Female	4 (100)
	Mental disorder diagnosis	
	BPD	1 (25)
	BPD and Major Depressive Disorder	1 (25)
	BPD and Bipolar Disorder	1 (25)
	BPD and Anorexia Nervosa Disorder	1 (25)

**Table 3:** Skill scores on the OTSM questionnaire.

	Family 1		Family 2		Family 3	Family 4	
	Couple	Daughter	Mother	Father	Mother	Mother	Father
Knowledge about BPD	8	9	8	7	8	9	8
Identification and management of emotions	7	8	7	6	7	9	7
Awareness of your family member’s emotions	8	8	7	8	6	9	7
Usefulness of Acceptance skills	10	9	10	10	9	10	10
Usefulness of Validation skills	9	9	10	10	8	10	9
Ability to validate your family member	9	8	9	9	9	9	9
Usefulness of Management of problems	9	10	10	10	9	10	9
Ability to manage problems with your family member	7	8	9	8	7	8	9

**Table 4:** Satisfaction and acceptance of FC on the OTSM questionnaire.

	Family 1		Family 2		Family 3	Family 4	
	Couple	Daughter	Mother	Father	Mother	Mother	Father
Program is logical	9	10	8	8	10	10	10
Satisfaction with the program	10	10	9	9	9	10	9
You would recommend the program	10	10	10	10	10	7	9
Usefulness of program and expectations	9	9	9	9	10	10	10
